# Emerging Roles of miRNAs in Brain Development and Perinatal Brain Injury

**DOI:** 10.3389/fphys.2019.00227

**Published:** 2019-03-28

**Authors:** Kenta Hyeon Tae Cho, Bing Xu, Cherie Blenkiron, Mhoyra Fraser

**Affiliations:** ^1^Department of Physiology, Faculty of Medical Health and Sciences, University of Auckland, Auckland, New Zealand; ^2^Departments of Molecular Medicine and Pathology, Faculty of Medical Health and Sciences, University of Auckland, Auckland, New Zealand

**Keywords:** perinatal, development, brain injury, miRNAs, biomarkers, exosomes, therapies

## Abstract

In human beings the immature brain is highly plastic and depending on the stage of gestation is particularly vulnerable to a range of insults that if sufficiently severe, can result in long-term motor, cognitive and behavioral impairment. With improved neonatal care, the incidence of major motor deficits such as cerebral palsy has declined with prematurity. Unfortunately, however, milder forms of injury characterized by diffuse non-cystic white matter lesions within the periventricular region and surrounding white matter, involving loss of oligodendrocyte progenitors and subsequent axonal hypomyelination as the brain matures have not. Existing therapeutic options for treatment of preterm infants have proved inadequate, partly owing to an incomplete understanding of underlying post-injury cellular and molecular changes that lead to poor neurodevelopmental outcomes. This has reinforced the need to improve our understanding of brain plasticity, explore novel solutions for the development of protective strategies, and identify biomarkers. Compelling evidence exists supporting the involvement of microRNAs (miRNAs), a class of small non-coding RNAs, as important post-transcriptional regulators of gene expression with functions including cell fate specification and plasticity of synaptic connections. Importantly, miRNAs are differentially expressed following brain injury, and can be packaged within exosomes/extracellular vesicles, which play a pivotal role in assuring their intercellular communication and passage across the blood–brain barrier. Indeed, an increasing number of investigations have examined the roles of specific miRNAs following injury and regeneration and it is apparent that this field of research could potentially identify protective therapeutic strategies to ameliorate perinatal brain injury. In this review, we discuss the most recent findings of some important miRNAs in relation to the development of the brain, their dysregulation, functions and regulatory roles following brain injury, and discuss how these can be targeted either as biomarkers of injury or neuroprotective agents.

## Introduction

MicroRNAs (miRNAs) are a class of endogenous small single-stranded non-protein coding RNA molecules (20–24 nucleotides), often phylogenetically conserved, which play a critical role in the control of gene expression at the post-transcriptional level. Specifically, miRNAs mainly function post-transcriptionally by binding to the 3′ untranslated region (3′UTR) of target messenger RNAs (mRNA) and induce mRNA degradation or translational repression (Bartel, 2009). In addition to their repressive role there is considerable evidence to support post-transcriptional stimulation of gene expression by miRNAs either in specific situations by direct or indirect mechanisms (Vasudevan, 2012).

Given their abundance in the central nervous system (CNS) and their specific patterns of expression within all of the major cell types during development (Sempere et al., 2004; Cao et al., 2006; Cherubini et al., 2006; Narayan et al., 2015), it is unsurprising that a number of miRNAs have emerged as potential regulators of CNS development and homeostatic function and under pathological conditions of hypoxia-ischemia, as mediators of neuroinflammation and neurodegeneration (Bhalala et al., 2013; Moon et al., 2013). Investigation of the possible relationships between miRNAs and their importance to the developing brain, however, remain in its infancy, since the majority of studies have not biologically validated the effects of miRNAs beyond the predicted mRNA targets. Nevertheless, a growing body of studies have demonstrated a critical role of miRNAs in the maturation of oligodendrocytes and myelin formation including the pathophysiology of hypoxia-ischemia-induced brain injury in the developing brain (Barca-Mayo and Lu, 2012; Fitzpatrick et al., 2015; Galloway and Moore, 2016; Su et al., 2016). In relation to the latter, it is presently unknown whether the roles of specific miRNAs or their profiles differ in response to injury with increasing gestational age. However, it is plausible that differences do indeed exist given their importance developmentally and since the neuropathology of brain injury differs as a function of gestational age. Among term infants the spectrum of injuries is dominated by selective necrosis, accompanied by parasagittal cerebral injury involving the paracentral cerebral cortex and associated white matter and represents a watershed injury in a vascular distribution (Ferriero, 2016; Kinney and Volpe, 2018b). In contrast, preterm infants born between 23 and 32 weeks gestation are at greatest risk of injury to the cerebral white matter. Depending on the severity of the insult, the spectrum of white matter injury in the preterm population can differ markedly. In its most severe form, all cell types are affected including oligodendrocytes, glia and axons resulting in focal cystic necrotic lesions (periventricular leukomalacia) forming within regions of the periventricular white matter adjacent to the lateral ventricular wall, which can extend into the centrum semiovale and the subcortical white matter (Back et al., 2002; Kinney, Volpe, 2018a). Milder forms are typically of a diffuse non-cystic variety and are now the most common type of injury observed in the preterm population. Moreover, the predominant pathology underlying diffuse white matter injury in the preterm infant is loss and subsequent arrested differentiation of pre-myelinating oligodendrocyte progenitors, (Volpe et al., 2011; Buser et al., 2012; Back and Miller, 2014; van Tilborg et al., 2016) which results in reduced brain myelination and potentially could be an avenue for miRNA targeted therapy.

In addition to the aforementioned role of miRNAs in the pathophysiology of perinatal brain injury evidence now suggests CNS cells secrete stable miRNAs into the plasma, which are bound to protein, HDL, or packaged within exosomes/microvesicles following stroke (Rao et al., 2013; Chen et al., 2015; Mondello et al., 2018). As their release is intimately related to genomic changes in the brain, they have immense potential as biomarkers of perinatal brain injury and may lead to early diagnosis, thereby allowing early implementation of treatment. This section will review emerging concepts associated with miRNA control of brain development and discuss their connection to perinatal brain injury impacted by inflammation and hypoxia-ischemia and those, which may serve as potential diagnostic biomarkers of injury and therapeutic targets.

## miRNAs in CNS Development

**FIGURE 1:**
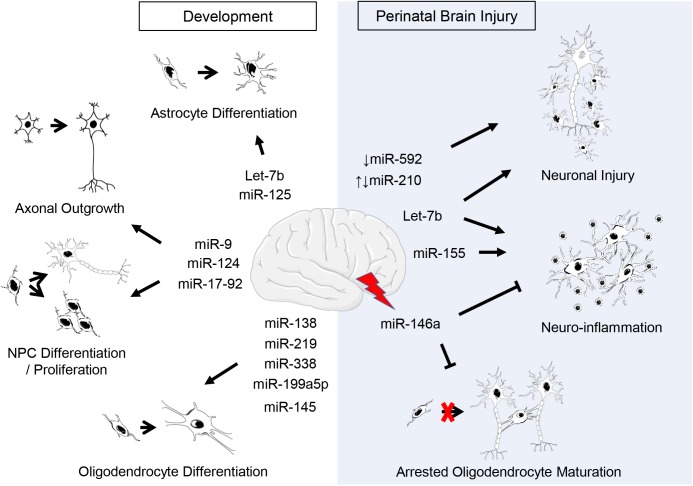
Putative roles of miRNAs in CNS development and perinatal brain injury. Listed are miRNAs covered in this review. *Supporting citations are reviewed in text and summarised below*. **Development:** Let-7b and miR-125 are regulators of astrocyte differentiation (Shenoy et al., 2015). miR-17-92 cluster is involved in neural progenitor cell (NPC) proliferation (Bian et al., 2013) and axonal growth (Zhang et al., 2013). miR-124 regulates neurogenesis (Krichevsky et al., 2006; Makeyev et al., 2007) and axonal growth of retinal ganglion cells (He et al., 2018). miR-9 is implicated in the regulation of NPC differentiation and proliferation (Krichevsky et al., 2006; Radhakrishnan and Alwin Prem Anand, 2016) as well as axonal development and neuronal migration (Dajas-Bailador et al., 2012; Otaegi et al., 2011). miR-219 (Dugas et al., 2010; Shin et al., 2009), miR-338 (Zhao et al., 2010), miR-138 (Dugas et al., 2010), miR-199a-5p (Letzen et al., 2010), miR-145 (Letzen et al., 2010) regulate oligodendrocyte (OL) differentiation. **Perinatal brain injury:** The downregulation of miR-210 is associated with an increase in neuronal apoptosis following hypoxic-ischemic injury (Qiu et al., 2013b) and the upregulation of miR-210 exacerbates cortical injury (Ma et al., 2016; Wang et al., 2017). Post-injury, miR-146a negatively regulates inflammation (Gaudet et al., 2018; Omran et al., 2012) and promotes oligodendrogenesis (Liu et al., 2017). Silencing or inhibition of miR-155 ameliorates inflammation post-injury (Ashhab et al., 2013; Caballero-Garrido et al., 2015; Pena-Philippides et al., 2016; Roitbak, 2018). Let-7b released from neurons and immune cells following injury exacerbates neuronal cell death and induces neuroinflammation (Lehmann et al., 2012; Mueller et al., 2014). The downregulation of miR-592 following hypoxic-ischemic injury induces apoptosis in hippocampal neurons (Sun et al., 2018).

Development of the mammalian CNS involves a series of intricately coordinated events that requires precise spatial and temporal control of gene expression at both the transcriptional and translational levels (Taverna et al., 2014; Gotz et al., 2016). As previously mentioned, the brain has an abundance of miRNAs; many are specific to a given cell lineage or cell type with some being shown to vary dynamically within the brain both prior to and after birth, suggesting a need for different miRNAs throughout development (Lagos-Quintana et al., 2003; Miska et al., 2004; Bak et al., 2008; Smith et al., 2010; Podolska et al., 2011; Ziats and Rennert, 2014; Chen and Qin, 2015). The interplay between miRNAs and their target mRNAs have a critical regulatory role during neural development, from early neurogenesis to synaptogenesis as well as maintenance of neural function (Figure 1) (Davis et al., 2015). miRNAs interact mainly through downregulation of expression of both intrinsic and extrinsic factors and activities of cell-specific signaling mechanisms, and therefore regulate the establishment and maintenance of cell fate specification and differentiation of neural stem cells and neurogenic niches (Shi et al., 2010; Brett et al., 2011; Barca-Mayo and Lu, 2012).

### miRNAs in Neuronal Cortical Development

The biological importance of miRNAs in neural development was first demonstrated by conditional knockout experiments of enzymes involved in miRNA biogenesis (Bernstein et al., 2003). The double-stranded RNA (dsRNA) nuclease Dicer is essential to this process (Petri et al., 2014). In mice, targeted ablation of the *Dicer1* gene affects brain development including impaired cortical neuron migration, microcephaly, and precursor differentiation in the spinal cord (Davis et al., 2008; De Pietri Tonelli et al., 2008). However, such studies do not readily assign roles for specific miRNAs since a deficiency in Dicer will affect the full complement of miRNAs in the targeted cells. Moreover, knockouts of specific miRNAs are often complicated, since bioinformatics analyses predict hundreds of targets for mammalian miRNAs, and it seems likely that many are indeed true targets (Lewis et al., 2003; Lim et al., 2005).

Despite these drawbacks, valuable information has been deemed regarding numerous miRNAs during brain development through loss-of-function and gain-of-function experiments (Figure 1). Evidence suggests that miRNAs play an important role in cortical development. For example, the miR-17-92 cluster, together with its paralogs miR-106a-363 and miR-106b-25, is required for appropriate development of embryonic fetal cells (Suh et al., 2004; Ventura et al., 2008). It consists of six miRNAs, processed from a common precursor transcript and grouped in four subfamilies, miR-17, miR-18, miR-19, and miR-92 (Ventura et al., 2008; Bian et al., 2013). A role for miR-17-92 in proliferation has been suggested since phosphatase and tensin homolog (*PTEN*; tumor suppressor gene) is one of its targets (Concepcion et al., 2012). Further, functional role studies have revealed that overexpression of the miR-17-92 cluster in axons of embryonic cortical cells modulates PTEN protein levels and increases axonal growth (Zhang et al., 2013). To confirm additional roles knockout studies of the miR-17-92 cluster and its paralogs have demonstrated an essential role of the miR-17-92 cluster in controlling expansion of neural stem cells and radial glial cells, and transition to intermediate progenitors, which are critical for normal cortical development and function (Bian et al., 2013). Moreover, knockout of miR-17-92 was associated with an upregulation of miR-17-92 target RNAs, *PTEN* and T-box transcription factor Eomes/*Tbr2* (*Tbr2*; a key regulator of neurogenesis in the SVZ), resulting in an increase in intermediate progenitors and suppression of cortical radial glial cells, respectively (Bian et al., 2013).

miR-124, the most abundant miRNA in the brain, is another well-studied regulator of neurogenesis, whose expression increases with commencement of neural differentiation and peaks in mature neurons (Krichevsky et al., 2006; Makeyev et al., 2007; Visvanathan et al., 2007; Cheng et al., 2009; Maiorano and Mallamaci, 2009; Ponomarev et al., 2011; Sanuki et al., 2011; Åkerblom et al., 2012; Sun et al., 2015). Targets of miR-124 include protein jagged-1 (Jag-1), Sry-type high mobility group box 9 (Sox9; involved in adult neurogenesis) and *DLX2* (transcription factor regulating neuronal subtype specification) (Cheng et al., 2009; Liu et al., 2011). Inhibition *in vivo* of miR-124 blocks neurogenesis resulting in a switch to gliogenesis, specifically inducing formation of ectopic astrocytes in the olfactory bulb derived from the subventricular zone (Åkerblom et al., 2012). Furthermore, overexpression experiments both *in vivo* and *in vitro* suggest that miR-124 plays a role in neural fate specification (Smirnova et al., 2005; Krichevsky et al., 2006; Silber et al., 2008; Åkerblom et al., 2012; Xia et al., 2012; Akerblom and Jakobsson, 2014) and most recently promotes axon growth of retinal ganglion cells differentiated from retinal stem cells (He et al., 2018).

Similarly, miR-9, a neuronal specific miRNA, with a prominent role in development, has also been implicated in the regulation of whether neural precursors will adopt a neuronal or glial fate (Krichevsky et al., 2006; Radhakrishnan and Alwin Prem Anand, 2016). miR-9 is highly expressed within the brain, primarily within neural precursors where it controls neural stem cell numbers (Delaloy et al., 2010; Akerblom et al., 2013; Coolen et al., 2013). Overexpression of miR-9 negatively regulates proliferation and accelerates neural differentiation through suppression of the orphan receptor TLX (human homolog of the tailless gene; also known as nuclear receptor subfamily 2, group E member 1 [Nr2e1]) suggesting that TLX and miR-9 participate in a feedback regulatory loop (Zhao et al., 2009). miR-9 is also involved in cortical axonal development via its target, microtubule-associated protein 1b (*Map1b*) (Dajas-Bailador et al., 2012). Furthermore, neuronal migration and outgrowth is also controlled by miR-9 through its interaction with forkhead transcription factors 1 and 2 (*Foxp1* and *Foxp2*) (Otaegi et al., 2011; Clovis et al., 2012).

### miRNAs in Oligodendrocyte Development

Due to the critical roles of miRNAs in neurogenesis, it is unsurprising that miRNAs have also emerged as important regulators of oligodendrocyte development (Figure 1). Microarray analysis of miRNA profiles in normal CNS development and *Dicer1* knockout models have identified miR-219 as a crucial regulator of oligodendrocyte differentiation (Shin et al., 2009; Dugas et al., 2010; Zhao et al., 2010). miR-219 is highly expressed in the white matter areas of the brain and expression persists in mature oligodendrocytes (Dugas et al., 2010). Its mechanism of action is via direct repression of expression of its predicted targets, namely platelet-derived growth factor receptor alpha (PDGFRα), SRY-box containing gene 6 (Sox6), forkhead box J3 (FoxJ3), and zinc finger protein 238 (ZFP238), all of which promote oligodendrocyte proliferation and inhibit oligodendrocyte differentiation (Barres et al., 1994; Stolt et al., 2006; Dugas et al., 2010). Transfecting purified oligodendrocytes with miR-219 mimic increases expression levels of early (2′,3′-cyclic nucleotide 3′-phosphodiesterase, CNP; myelin basic protein, MBP) and late (myelin oligodendrocyte glycoprotein, MOG) oligodendrocyte specific differentiation markers (Dugas et al., 2010; Zhao et al., 2010). Furthermore, addition of miR-219 mimic to oligodendrocyte progenitor cells lacking functional *Dicer1* expression and which display deficits in myelin gene expression (CNP, MBP, and MOG), markedly enhanced maturation and restored their expression levels to control transfected cells (Dugas et al., 2010; Zhao et al., 2010). Cumulatively, these data indicate that miR-219 is critical for the coordinated transition of oligodendrocyte progenitor cells to oligodendrocytes and subsequent myelin formation and thus may have potential as a therapeutic strategy to promote myelination following injury.

Other important regulators of oligodendrocyte progenitor differentiation are miR-338 and miR-138 (Lau et al., 2008; Dugas et al., 2010). miR-338 is equally as significant as miR-219 in controlling oligodendrogenesis and shares common targets notably Sox6 and Hes Family BHLH Transcription Factor 5 (Hes5); both of which are negative regulators of myelin gene expression (Liu et al., 2006; Stolt et al., 2006; Dugas et al., 2010; Zhao et al., 2010). Furthermore, miR-338 is upregulated in mature oligodendrocytes (Lau et al., 2008) and its overexpression increases oligodendrocyte differentiation (Zhao et al., 2010). However, the role of miR-138 is somewhat incongruous. While miR-138 expression is also elevated in oligodendrocyte precursors its impact on oligodendrocyte development is less significant than miR-219 and miR-338 (Dugas et al., 2010). In contrast to miR-219, oligodendrocyte progenitors induced to differentiate by miR-138 mimic, only express early oligodendrocyte differentiation markers (CNP, MBP) but not late differentiation markers (Dugas et al., 2010). Moreover, miR-138 inhibits Sox4 transcription factor, a repressor of oligodendrocyte maturation (Potzner et al., 2007; Yeh et al., 2013). Together these findings suggest that miR-138 may play a role in extending the period oligodendrocytes are maintained in the early phase of oligodendrocyte differentiation thereby providing a suitable time frame for terminally differentiating oligodendrocytes to myelinate neighboring axons.

Elegant studies by Letzen et al. (2010), using human embryonic stem cells to investigate miRNA expression profiles have revealed unique patterns of expression during the various stages of oligodendrocyte differentiation and maturation. Specifically, four main clusters of miRNA expression were identified encompassing the breadth of the oligodendrocyte lineage scheme (early, mid, and late progenitors and mature oligodendrocytes). Predicted targets of the top differentially expressed genes included myelin-associated genes namely chromosome 11 open reading frame 9 (*C11Orf9*), myelin gene regulatory factor (*MRF*), claudin-11 (*CLDN11*), myelin transcription factor 1-like (*MYTL1*), myelin-associated oligodendrocyte basic protein (*MOBP*), myelin protein zero-like 2 (*MPZL2*), and discoidin domain receptor tyrosine kinase 1 (*DDR1*). Of interest, the authors showed that within the top 10 differentially expressed miRNAs, spanning early to mid-oligodendrocyte progenitor stages, both miR-199a-5p and miR-145 were strongly biased to *C11Orf9*, a gene considered to play a critical role in oligodendrocyte maturation and myelin production.

Evidence discussed above, thus highlights the need to define the role of miRNAs in normal neurodevelopment since they may lay the foundations for novel miRNA-based therapies for preterm infants at risk of brain injury.

### miRNAs in Astroglial and Microglial Development

Within cells of the neural lineage, information on the function of miRNAs is predominately limited to neuronal and oligodendrocyte differentiation. Only a relatively few studies have been conducted to investigate the role of miRNAs in astrogliogenesis. This is somewhat surprising given astrocytes represent a major glial cell type in the CNS and are powerful homeostatic regulators of brain function (Giaume et al., 2010). Presumably, the difficulty encountered in isolating astrocyte progenitors *in vivo* has been a major constraint when investigating the functions of astrocyte miRNAs. However, in a recent study of glial progenitors induced to differentiate into astrocytes, deletion of all canonical miRNAs by conditional knockout of Dgcr8 (the RNA binding protein involved in processing of all canonical miRNAs) blocked astrocyte differentiation *in vitro* (Shenoy et al., 2015). Such results were also in keeping with Dicer-knockout studies of *in vivo* derived multipotent neural stem cells (Andersson et al., 2010). Furthermore, in the study conducted by Shenoy et al. (2015), let-7 and miR-125, operating through several targets, restored astrocyte differentiation. Additional studies of disruption of both astrogliogenesis and oligodendrogenesis with inhibition of miRNA formation in ventral spinal progenitors from Olig1^Cre^ – mediated Dicer conditional knockout mice provide further support for miRNAs role in gliogenesis (Zheng et al., 2010, 2012). It is also important to note that a recent study has provided unprecedented evidence of miRNA expression profiles of astrocytes isolated by laser capture microdissection from various regions within the human second trimester fetal brain (17–20 weeks gestation) and adult brain (24–76 years) with no discernible pathology (Rao et al., 2016). Regional differences were noted in these studies, as well as lower expression of miRNAs in fetal vs. adult white matter astrocytes and high expression of miRNAs in the fetal germinal matrix, which presumably is of relevance in pathological conditions.

Microglia are another major glial population. Depending on their location within the CNS, microglia can vary in morphology and density and have important functions in immune surveillance, mediating innate immune responses. In recent years there has been an exponential increase in investigations focussing on the function and regulation of microglia by intrinsic and extrinsic factors within the developing and adult brain under both normal and abnormal physiological conditions (Baburamani et al., 2014; Katsumoto et al., 2014; Nayak et al., 2014; Hagberg et al., 2015; Michell-Robinson et al., 2015; Reemst et al., 2016; Li et al., 2017; Tay et al., 2017; Thion et al., 2018). However, to date, Ponomarev et al. (2011, 2013) have performed the only studies thus far on the role of miRNAs in microgliogenesis within the CNS and have demonstrated that miR-124 is highly expressed in normal CNS-resident microglia, but absent in peripheral monocytes and macrophages. As discussed previously (see section “miRNAs in Neuronal Cortical Development”), miR-124 is also highly expressed in other regions of the CNS and is an important regulator of neurogenesis and neuronal differentiation through its regulation of neuronal gene expression.

Ponomarev et al. (2011, 2013) also showed that miR-124 is a key promoter of the quiescent state of microglia. By forced overexpression of miR-124 in macrophages they were able to demonstrate that miR-124 negatively modulates CCAAT/enhancer-binding protein-α (C/EBP-α) transcription factor, and its downstream target PU.1, resulting in their transformation from an activated to a quiescent phenotype (Ponomarev et al., 2011, 2013). Furthermore, knockdown of miR-124 in microglia and macrophages returned both cells into an activated state (Ponomarev et al., 2011). Thus, this supports a role for miR-124 in the maintenance of a resting phenotype through targeting of the CEBPα/PU.1 pathway and possibly is a way to establish an “alternative” activation (M2) phenotype in resident microglia as part of the reparative response to hypoxia-ischemia- or infection-related neuroinflammation (see section “miRNAs and Neuroinflammation”). Finally, it is apparent that there is a need to identify other candidate miRNAs who may participate in developmental regulation of astrocytes and microglia.

## Role of miRNAs in Perinatal Brain Injury

An extensive body of literature is now available to suggest dysregulation of miRNA biogenesis and their regulatory role is a common theme associated with the development of neurological injury and disorders from adult experimental models and patients (Dharap et al., 2009; Tan et al., 2009; Liu et al., 2010; Yuan et al., 2010; Bhalala et al., 2013; Eacker et al., 2013; Khanna et al., 2013; Moon et al., 2013; Wang and Yang, 2013; Ouyang et al., 2014). Accordingly, given this and evidence of miRNAs regulatory role during all stages of CNS development, there has been an emerging interest into the implications of miRNAs in perinatal brain injury (Figure 1).

### HypoxamiRs

Insults such as impaired oxygen delivery or hypoxia has the potential to elicit expression of a distinct group of miRNAs known as hypoxamiRs, that according to the miRbase database (Griffiths-Jones, 2006; Kozomara and Griffiths-Jones, 2014) are in excess of a 100. Importantly, the specific hypoxamiR signature in response to hypoxia varies according to cell type affected and physiological response (Kulshreshtha et al., 2007; Nallamshetty et al., 2013).

Hypoxic regulation of miR-210, considered to be the master hypoxamiR, was first identified by miRNA microarray over a decade ago (Kulshreshtha et al., 2007) and has been shown to be consistently upregulated under various hypoxic conditions (Huang et al., 2010; Chan et al., 2012). Indeed, multiple studies involving adult models of ischemic stroke have consistently shown that miR-210 induction is a feature of the hypoxic response (Jeyaseelan et al., 2008; Dharap et al., 2009; Qiu et al., 2013b; Liu et al., 2018; Meng et al., 2018). Furthermore, in terms of neurogenesis, studies are contradictory in relation as to whether miR-210 inhibition increases neurogenesis following ischemia (Zeng et al., 2014; Ma et al., 2016; Voloboueva et al., 2017). Such differences presumably relate to timing of miR-210 inhibition, since evidence points to a reduction in proliferation with early post-ischemic inhibition, whereas later it increases neurogenesis (Voloboueva et al., 2017).

Similar controversy exists in relation to the immature brain, as evidence from various neonatal stroke models suggest miR-210 may play either a protective or a detrimental role (Ma et al., 2016). For instance, Qiu et al. (2013a), using a PC12 cell model of oxygen glucose deprivation reported that miR-210 reduced PC12 cell death. The same group demonstrated that in PD (postnatal day; day of birth = postnatal day 0) 7 neonatal rats, miR-210 expression is downregulated in response to hypoxia-ischemia in association with increased brain edema (Qiu et al., 2013b) and that pretreatment with miR-210 mimic significantly reduced edema indicating a possible protective role in response to ischemia.

In contrast, Ma et al. (2016) reported that miR-210 is upregulated following a 2.5 h period of hypoxia-ischemia in PD10 neonatal rats. Furthermore, they demonstrated that miR-210 directly targets the 3′UTR region of the glucocorticoid receptor (GR) in the neonatal rat brain and down regulates GR protein following hypoxia-ischemia resulting in increased susceptibility to injury. In the same study, silencing of miR-210 by intracerebroventricular (ICV) administration of complementary locked nucleic oligonucleotides (miR-210-LNA, miR-210 inhibitor), 4 h after hypoxia-ischemia, significantly ameliorated neuronal injury and infarct size in association with a reduction in brain miR-210 levels. Interestingly, intranasal administration of miR-210-LNA under the same conditions resulted in similar effects. Additional studies by Ma et al. (2017), revealed ICV administration of miR-210 mimic in neonatal rats 48 h prior to hypoxic-ischemic injury compromised blood–brain barrier integrity by suppressing junction proteins, thus resulting in increased susceptibility to brain edema and immunoglobulin G (IgG) parenchyma leakage across the blood–brain barrier.

Finally, in a neonatal rat model of perinatal nicotine-sensitized hypoxic-ischemic brain injury, prior treatment with nicotine was associated with increased miR-210 expression, decreased brain-derived neurotrophic factor/tropomyosin receptor kinase B (BDNF/TRKB) protein expression, and increased susceptibility to hypoxic-ischemic injury (Wang et al., 2017). Moreover, ICV administration of miR-210-LNA 48 h before hypoxia-ischemia significantly decreased brain infarct size in both saline control and nicotine-treated cohorts to levels comparable. To conclude, since a number of verified and putative targets have been identified for miR-210 (Chan and Loscalzo, 2010), it is likely that there are contradictions found with respect to miR-210-specific effects as mentioned above. Such putative roles in relation to perinatal brain injury await corroboration that is more definitive.

### miRNAs and Oligodendroglial Response to Hypoxia-Ischemia

Studies highlighting miRNAs as key regulators of oligodendrocyte development may have significant clinical implications with respect to further understanding the pathogenesis of preterm hypoxia-ischemia brain injury, since loss and subsequent arrested differentiation of oligodendrocyte progenitors is a hallmark of injury. Presently, there is a paucity of information with regard to how miRNA expression contributes to critical events of oligodendrogenesis occurring in response to hypoxia-ischemia within the developing brain.

Recently, however, the role of miRNAs in perinatal hypoxia-ischemia has been evaluated in NG2 specific *Dicer1* knockout mice (Birch et al., 2014). Loss of Dicer within oligodendrocyte progenitors following hypoxia-ischemia increased both the number of mature oligodendrocytes and MBP expression, which was associated with improved motor co-ordination performance. Furthermore, in the same study, miRNA profiling within lesion sites of wild-type mice, demonstrated delayed but significant increases in miR-138 and miR-338, 7 days following hypoxia-ischemia. These findings are difficult to resolve since *Dicer1* knockout would normally result in myelin loss and since miR-138 and miR-338 increases with oligodendrocyte differentiation, which was shown to be impaired with hypoxia-ischemia. The authors, however, proposed that mature miRNAs upregulated in response to hypoxia-ischemia may increase oligodendrocyte progenitor proliferation rate and thus decrease inversely differentiation. Further studies are required to address the roles of these miRNAs in this model of perinatal hypoxia-ischemia.

### miRNAs and Neuroinflammation

Inflammatory responses play key roles in the regulation of neurodevelopment, neurodegeneration and injury. Due to their capacity to regulate simultaneously a cascade of different genes, miRNAs are well placed as key regulators of neuroinflammation and their dysfunction is equally recognized as contributing to adverse neuroinflammatory processes (Su et al., 2016). Depending upon the target mRNAs and stimulant involved, miRNAs can exhibit functions that are either pro-inflammatory, anti-inflammatory, and/or mixed immunomodulatory in nature.

The most notable of these miRNAs are miR-155 and miR-146a. While miR-155 has both pro- and anti-inflammatory functions (Duan et al., 2016), it is widely considered to be the most potent pro-inflammatory miRNA (Gaudet et al., 2018), and recognized as a key regulator of microglial-mediated immune responses (Cardoso et al., 2012; Butovsky et al., 2015). In the context of adult cerebral ischemia, there is substantial evidence that silencing or inhibition of miR-155 ameliorates the damaging effects of neuroinflammation (Liu et al., 2010; Caballero-Garrido et al., 2015; Pena-Philippides et al., 2016; Roitbak, 2018). Importantly, miR-146a, a negative regulator of inflammation, is characteristically upregulated in the pathogenesis of various neurological conditions (Gaudet et al., 2018) and considered to play a key role in the regulation of cell survival responses by negative regulation of Toll-like receptor 4 (TLR4) through targeting tumor necrosis factor (TNF) receptor-associated factor 6 (*TRAF6*) and interleukin-1 receptor-associated kinase 1 (*IRAK1*) genes in innate and adaptive immune cells (Taganov et al., 2006; Baltimore et al., 2008; Mann et al., 2017). In addition, miR-146a is a key of regulator of oligodendrogenesis both in the normal (Galloway and Moore, 2016) and ischemic brain (Liu et al., 2017) and a negative-feedback regulator of astrocyte-mediated inflammation (Taganov et al., 2006; Iyer et al., 2012).

While growing evidence has revealed that several miRNAs including miR-155 and miR-146a regulate the extent and timing of TLR responses and innate immune pathways (Taganov et al., 2006; O’Connell et al., 2007; Nahid et al., 2011; O’Neill et al., 2011; Quinn and O’Neill, 2011; Lehmann et al., 2012), little is known about their roles in modulation of neuroinflammation within the immature brain following injury. A study to examine the effect of inflammation on epileptogenesis revealed that miR-146a is upregulated in both a PD11 neonatal rat pilocarpine model of mesial temporal lobe epilepsy (MTLE) and children with MTLE and suggest miR-146a modulates the inflammatory response triggered by interleukin-1β (IL-1β) by inhibiting its expression level thus supporting a neuroprotective role for miR-146a (Omran et al., 2012). Further, studies from the same group and animal model revealed that miR-155 and TNF-alpha (TNF-α) is upregulated in seizure-related acute and chronic stages of MTLE (Ashhab et al., 2013). Similar dysregulation was also observed in children with MTLE, thus supporting a role for miR-155 and TNF-α in the development of seizure susceptibility in the immature brain.

Additional support for miR-146a pro-survival functions is also provided by a recent study conducted in PD1 neonatal rats exposed to hypoxia, in conjunction with BV-2 cells (Zhou et al., 2015). The authors showed that treatment with thymosin β4 (Tβ4), a major actin-sequestering protein, known to reduce inflammation and stimulate remyelination after neurological injury (Morris et al., 2010; Xiong et al., 2012), inhibited microglial activation and was associated with *in vitro* upregulation of miR-146a expression (Zhou et al., 2015). Interestingly, Tβ4 upregulation of miR-146a has been shown to promote oligodendrocyte differentiation and suppression of TLR pathways, thus adding to its therapeutic implications (Santra et al., 2014). Furthermore, lipopolysaccharide (LPS) *in vitro* stimulation of newborn cord blood results in upregulation of miR-146a expression in monocytes implicating its involvement in neonatal innate immune responses (Lederhuber et al., 2011). Similarly, studies of miRNA expression profiles from leukocytes isolated from newborn whole cord blood following LPS *in vitro* stimulation show a total of 85 miRNAs are differentially expressed of which several are proposed to modulate TLR inflammatory pathways (Chen J. et al., 2014). As previously discussed (see section “miRNAs in Astroglial and Microglial Development”), miR-124 is another example of a miRNA that regulates CNS inflammation and is highly expressed in microglia and can reduce CNS inflammation through promotion of microglia quiescence via the C/EBP-α-PU.1 pathway. Consequently, overexpression of miR-124 in microglia can induce a switch to M2 polarization, shown by expression of interleukin-10 (IL-10) and transforming growth factor β (TGF-β) (Ponomarev et al., 2011, 2013). Indeed, miR-124 could potentially become a powerful therapeutic strategy for alleviating brain injury in the perinatal period.

Let-7b, a highly abundant miRNA (Pena et al., 2009) and regulator of gene expression in the CNS, released from injured neurons and immune cells, has been demonstrated to exacerbate CNS injury through activation of TLR7 and induce neurodegeneration through neuronal TLR7 (Lehmann et al., 2012). Furthermore, ICV administration of an antagomir to let-7f, another let-7 family member, has been demonstrated to reduce cortical and striatal infarcts in an adult rat stroke model and be preferentially expressed in microglia within the ischemic boundary zone (Selvamani et al., 2012). Recently Mueller et al. (2014), demonstrated that a synthetic peptide analogous to the mammalian preimplantation factor (PIF) secreted by embryos and which is present in the maternal circulation during pregnancy inhibits let-7 miRNA biogenesis in both murine N2a neuroblastoma cells and RAW 264.7 macrophage cell lines. Using a PD3 neonatal rat hypoxic-ischemic brain injury model these authors then showed that subcutaneous administration of synthetic PIF 3 days after injury, significantly abolished the cortical volume reduction, neuronal loss and microgliosis associated with injury in this model 10 days after injury. Although the neuroprotective mechanism remains unclear these authors provided data to suggest that TLR4 may play an important role in synthetic PIF-induced let-7 repression and that KH-type splicing regulatory protein (KSRP) known to be involved with the biogenesis of the let-7 family of miRNAs and a mediator of mRNA decay (Repetto et al., 2012) may be an interacting cofactor involved with this process. Characterization of the specific signaling pathways activated is required to elucidate the significance of this potential pathway in mediating neuroprotection of the developing brain.

## miRNAs as Potential Biomarkers

There are numerous other brain-specific miRNAs known to play potentially crucial roles in the pathological processes of adult brain injury, whose roles in relation in perinatal brain injury have yet to be determined. Nevertheless, recent studies have focussed on identification of several miRNAs as potential biomarkers of perinatal brain injury to enable early diagnosis of the severity of injury (Table 1).

**Table 1:** Potential miRNA biomarkers of perinatal hypoxia-ischemia.

miRNA biomarkers of perinatal HI	Studied cohorts/subjects	Sampling source	Techniques employed for miRNA detection	References
↓ miR-374a	70 newborn infants	Umbilical cord blood	Microarray, qRT-PCR	Looney et al., 2015
	18 controls, 33 with perinatal asphyxia in the absence of HIE, 19 infants with HIE			
↑ miR-374a	13 newborn piglets	Plasma sample	qRT-PCR	Garberg et al., 2016
↑ miR-210	5 controls, 8 with transient global HI			
↑ miR-210	P10 rat pups	Ipsilateral hemisphere	qRT-PCR	Ma et al., 2016
	4 controls, 4 with right carotid ligation induced HI			
↑ miR-21	78 newborn infants	Serum sample	qRT-PCR	Chen and Yang, 2015
	29 controls, 49 with asphyxia			
↑ miR-210	24 infants	Maternal whole blood sample	qRT-PCR	Whitehead et al., 2013
↑ miR-424	12 controls, 12 severely growth restricted preterms			
↑ miR-21				
↑ miR-199a				
↑ miR-20b				


*HIE: hypoxic-ischemic encephalopathy, HI: hypoxia-ischemia, qRT-PCR: quantitative real-time polymerase chain reaction.*

Studies, conducted by Looney et al. (2015), involving the analysis of umbilical cord blood miRNA profiles from a cohort of 70 newborn infants [18 controls, 33 with perinatal asphyxia in the absence of hypoxic ischemic encephalopathy (HIE), and 19 infants with HIE analysis], have revealed 70 miRNAs that are differentially expressed with injury. Notably, miR-374a was significantly downregulated in infants with electroencephalographic (EEG) confirmed HIE vs. controls, and further substantiated by quantitative real-time PCR analysis. While no functional mechanism of action and pathways were confirmed, target analysis revealed specific pathways and biological processes associated with neurological injury. Further research from the same group identified several potential downstream targets of this miRNA, namely activin-A receptor type IIb (*ACVR2B*) (Looney et al., 2017). Despite the lack of confirmation of a significant increase in activin-A levels, as previously demonstrated in biological fluids following perinatal asphyxia and HIE (Florio et al., 2004; Florio et al., 2007; Douglas-Escobar and Weiss, 2012), significantly increased levels of ACVR2B were detected in infants with severe HIE. Of interest, however, is the recent demonstration by Dillenburg et al. (2018) that overexpression of Acvr2b in oligodendroglial lineage cells impairs Acvr2a-regulated oligodendrocyte differentiation and myelin formation, thus supporting the possibility of restoration of Acvr2a-mediated signaling as a strategy to combat perinatal white matter injury.

Aside from the above clinical investigation, a recent study of global hypoxia-ischemia in newborn piglets has also provided evidence to support circulating plasma miR-374a and the hypoxamiR miR-210 as potential biomarkers (Garberg et al., 2016). However, in contrast to Looney et al. (2015), these authors reported a significant upregulation of miR-374a 9.5 h after hypoxia-ischemia and noted that correlations were found between miR-374a and arterial pH, base excess and lactate levels over the study period. Since miR-374a is directly regulated by lactate dehydrogenase A with hypoxia (Wang et al., 2015), the authors concluded that miR-374a might play a role in metabolic adaptive responses to hypoxia-ischemia. Nevertheless, the increase in miR-210 is in congruence with previous studies under a hypoxic environment (Huang et al., 2010; Chan et al., 2012) and those observed by Ma et al. (2016) following hypoxia-ischemia in PD10 neonatal rats.

Other candidate miRNAs have been investigated, namely let7b, miR-29b, miR-124, miR-155, and miR-21 (Ponnusamy et al., 2016). Quantification of these miRNAs in dried blood spots, EDTA-blood, plasma and urine collected from a small cohort of newborns, failed to demonstrate significant differences with injury. However, miR-21, which is expressed in astrocytes, and been shown in adult plasma to be a potential early stage marker of acute cerebral infarction (Zhou and Zhang, 2014), was found to be elevated in serum of 49 neonates with HIE, thus providing support also for an early diagnosis biomarker of neonatal HIE (Chen and Yang, 2015).

Additional studies that warrant mention are preliminary studies conducted by Whitehead et al. (2013). An analysis was made of maternal whole blood expression levels of six miRNAs known to be associated with hypoxia in which fetuses had either experienced acute hypoxia during labor or chronic hypoxia associated with fetal growth retardation. Compared to gestational matched controls there was an upregulation of miR-210, miR-424, miR-21, miR-199a, and miR-20b. Furthermore, correlation with Doppler velocimetry assessments of hypoxia, confirmed the increase was associated with increased severity of hypoxia. The changes observed in miR-210 agree well with that of Ma et al. (2016) and those conducted in the piglet (Garberg et al., 2016) thus supporting the use as a maternally based biomarker of fetal hypoxia.

A caveat: while the field of miRNA research is constantly expanding one must appreciate that the endogenous source of miRNAs within available body fluids including, plasma, serum, urine and saliva can be from a diverse array of peripheral tissue cellular types including those of the brain, thus decreasing in essence the reliability of results. The other point to note is the problem of cell specification of miRNAs; the reality for many miRNAs as biomarkers in body fluids is that they may not be expressed exclusively within one particular cell type. In the last few years, however, since the identification of exosomes [cell-derived vesicles; typically ∼40–100 nm in diameter (Raposo and Stoorvogel, 2013)], as a carrier of protein, lipids, mRNAs and miRNAs, with an important role in cell–cell-communication, there has been intense interest into whether brain-derived exosomes could serve better as biomarkers in the clinical diagnosis and management of brain injury (Valadi et al., 2007; Ludwig and Giebel, 2012; Patz et al., 2013; Taylor and Gercel-Taylor, 2014; Werner and Stevens, 2015). Additionally, unlike free circulating miRNAs, miRNAs are inherently enriched and stable within exosomes (Cheng et al., 2014). In the adult, increased levels of exosomes are released from cells following stroke and traumatic brain injury (Patz et al., 2013; Chiva-Blanch et al., 2016). Based on current information, it is apparent that brain-derived exosomes can traverse across the blood–brain barrier following injury. Their presence in the peripheral circulation places them in an ideal position to provide an informative platform for real-time assessment of newborns who have sustained brain injury or those who are at risk of adverse outcomes and to spearhead therapeutic discovery. However, such enthusiasm must be tempered by the harsh reality that proportionally brain-derived exosomes may represent only a small population of circulating exosomes whereas the contribution from peripheral sources may be relatively high in comparison. Although still in its infancy, platforms that employ microscale structures (e.g., microfluidics or acoustofluidics) (Contreras-Naranjo et al., 2017; Guo et al., 2018; Hisey et al., 2018; Li et al., 2018; Wu et al., 2018) and high-throughput phenotypic and functional analyses (e.g., advanced imaging flow cytometry) (Mastoridis et al., 2018) could circumvent this problem. If advanced to such a degree as to provide a rapid and effective means to selectively sort and detect brain-derived exosomes at a nanoscale level they would be of significant value for clinical evaluation.

In the adult, only a few clinical studies have been performed to assess the potential of exosomal-derived miRNAs as biomarkers of acute brain injury. Studies by Chen et al. (2017) demonstrated there was an association between increased circulating levels of exosomal miR-223 and acute ischemic stroke occurrence, stroke severity, and short-term outcomes. Furthermore, studies conducted by Ji et al. (2016) showed that serum exosomal miR-9 and miR-124 levels were positively associated with adverse scores of acute stroke injury, infarct volumes, and serum concentrations of the pro-inflammatory cytokine, interleukin-6 (IL-6). Currently, however, little is known of the usefulness of exosomal-derived miRNAs in the diagnosis of perinatal brain injury. Nevertheless, in a recent study conducted to investigate whether exosomal protein biomarkers would be valuable in the diagnosis of brain injury and assessment of the effectiveness of hypothermia, it was shown that neutral or decreasing serum neuronally derived exosomal synaptopodin protein levels occurred in neonates with abnormal neuroimaging scores (Goetzl et al., 2018).

There is also a growing interest as to the regenerative utility of exosomal-derived contents with and without loading with therapeutics (Doeppner et al., 2015; Luarte et al., 2016; Xiong et al., 2017; Kim et al., 2018). Several studies have also documented the neuron healing and protective abilities of stem cell derived exosomes (Lee et al., 2013; Zhang et al., 2015b; Long et al., 2017; Willis et al., 2017). Importantly, studies recently reported highlight the regenerative potential of mesenchymal stem cell (MSC)-derived extracellular vesicles in a preterm fetal sheep model of hypoxia-ischemia (Ophelders et al., 2016). In these studies, systemic administration to the fetus of MSC-extracellular vesicles resulted in improved brain activity namely a reduction in duration and number of seizures. Finally, as discussed later in Section “*In vivo* Evidence,” it warrants mentioning that because exosomes can potentially act as a therapeutic delivery system they hold great promise in revolutionizing the way we can effectively treat perinatal brain injury.

## miRNAs as Potential Therapeutic Targets

**FIGURE 2:**
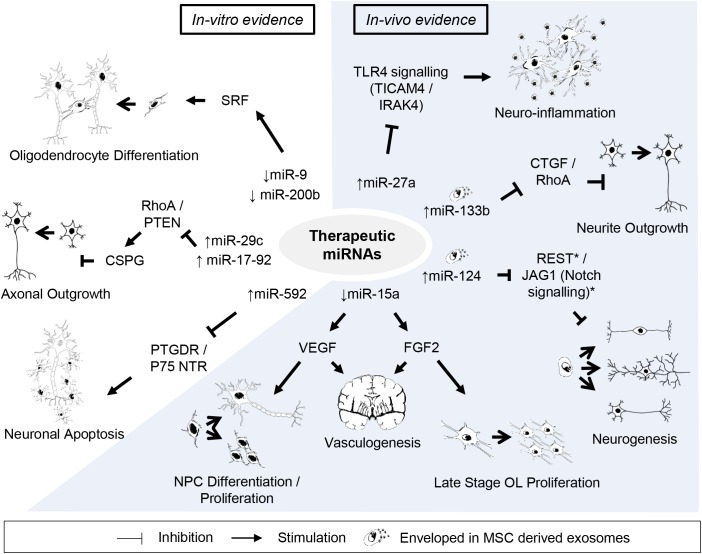
Potential candidates of miRNA-based therapy against perinatal hypoxic-ischemic brain injury. Listed are miRNAs and their downstream targets supporting potentially beneficial outcomes after perinatal hypoxic-ischemic brain injury. *Supporting citations are reviewed in text and summarised below*. ***In-vitro* evidence:** Downregulation of miR-9 and miR-200b mediates serum response factor (SRF) induced oligodendrocyte progenitor cell (OPC) differentiation (Buller et al., 2012). miR-29c (Yi Zhang et al., 2015) and miR-17-92 cluster (Zhang et al., 2013) attenuates the inhibitory effect of chondroitin sulphate proteoglycans (CSPG) on axonal regrowth by stimulating ras homolog family member A (RhoA) and phosphate and tensin homolog (PTEN) protein levels. miR-592 attenuates the activation of pro-apoptotic signalling and neuronal death by targeting the prostaglandin D2 receptor (PTGDR) and neurotrophin receptor (NTR) p75 (Irmady et al., 2014; Sun et al., 2018). ***In-vivo* evidence:** miR-27a reduces TLR4-mediated inflammation (Li et al., 2015). Downregulation of miR-15a expression promote vasculogenesis by stimulating neurotrophic factors, fibroblast growth factor 2 (FGF2) and vascular endothelial growth factor (VEGF) (Yin et al., 2012), which can further support late stage oligodendrocyte (OL) proliferation/migration (Shindo et al., 2016) and neural progenitor cell (NPC) differentiation/proliferation (Teng et al., 2008), respectively. Mesenchymal stem cell (MSC) derived exosomal transfer of miR-133b enhanced neurite outgrowth by inhibiting connective tissue growth factor (CTGF) and RhoA expression (Xin et al., 2012, 2013). Exosome mediated neuronal delivery of miR-124 induces neurogenesis (Yang et al., 2017) speculatively via Usp14-dependent REST degradation (Doeppner et al., 2013) and inhibition of the JAG/Notch signalling pathway (Liu et al., 2011).

In alliance with the discovery of miRNAs as functional regulators of cell development, miRNAs have also been shown to orchestrate a variety of critical signaling pathways involved in injury progression and recovery (Shi et al., 2010; Gaudet et al., 2017). Given the recent demonstration that modulation of miRNA expression occurs following a hypoxic-ischemic insult in the developing brain, several therapeutic targets have emerged (Figure 2).

### *In vitro* Evidence

*In vitro* studies have provided insights into the therapeutic potential of miRNAs that regulate reparative processes following a hypoxic-ischemic insult. For example, stroke-induced downregulation of miR-9 and miR-200b expression in the ischemic white matter region mediated serum response factor (SRF) induced differentiation of oligodendrocyte precursor cells (OPCs) into oligodendrocytes (Buller et al., 2012). Accordingly, *in vitro* overexpression of miR-9 and miR-200b suppressed SRF expression and inhibited OPC differentiation (Buller et al., 2012). Upon validation *in vivo*, the inhibition of miR-9 and miR-200b following injury may indicate a potential therapeutic strategy in the future given that myelination disturbances in the cerebral white matter represent a hallmark of perinatal brain injury (Pandit et al., 2013; Back and Miller, 2014).

Chondroitin sulfate proteoglycans (CSPGs) are well-characterized inhibitory extracellular matrix molecules expressed by reactive astrocytes, endothelial and oligodendrocyte progenitor cells that inhibit axonal regeneration after injury and are associated with adverse neurological outcome in preterm infants (McKeon et al., 1999; Jones et al., 2002, 2003; Chow et al., 2005). *In vitro* overexpression of miR29c and miR-17-92 cluster in embryonic cortical neurons has been shown to attenuate the inhibitory effect of CSPG by stimulating intrinsic axonal signals, suppressing Ras homolog gene family, member A (RhoA) and phosphate and tensin homolog (PTEN) protein levels, thereby promoting axonal outgrowth (Park et al., 2008; Zhang et al., 2013, 2015a). Thus, the potential loss or impaired axonal growth observed in focal necrotic white matter injury in the preterm brain could be feasibly targeted (Riddle et al., 2012).

miR-592 was originally suggested as a possible target for promoting cell apoptosis in various cancers (Liu M. et al., 2015; Liu Z. et al., 2015; Fu et al., 2016). Unsurprisingly, recent studies have also supported its regulatory role of cell death following cerebral ischemic injury (Irmady et al., 2014; Sun et al., 2018). In two studies carried out by Irmady et al. (2014) and Sun et al. (2018), both authors observed reduced expression of miR-592 following cerebral ischemic injury in the hippocampus of neonatal and juvenile mice, respectively. Concordantly, overexpression of miR-592 in cultured hippocampal neurons attenuated the activation of pro-apoptotic signaling and cell death (Irmady et al., 2014; Sun et al., 2018). The mechanism underlying this protective mechanism speculates the multi-functional role of miR-592. Sun et al. (2018) demonstrated that miR-592 affords neuroprotection by selectively targeting prostaglandin D2 receptor (PTGDR) and inhibiting prostaglandin D2 (PGD2)-DP signaling, an inflammatory pathway involving the release of glutamate (Weaver-Mikaere et al., 2013). Irmady et al. (2014), on the other hand revealed that vector mediated transfection of miR-592 in embryonic hippocampal neurons attenuated the level of neurotrophin receptor (NTR) p75 induced by ischemic injury and subsequent apoptotic cell death. The NTR p75 is a member of the TNF receptor superfamily closely implicated with neuronal apoptosis following experimental perinatal brain injury (Volosin et al., 2006; Griesmaier et al., 2010). Given the prospective dual anti-apoptotic mechanism of miR-592, results of future *in vivo* studies are eagerly awaited.

### *In vivo* Evidence

miR-27 is a potential regulator of cortical neuronal apoptosis whose expression in embryonic mouse cerebral cortices is attenuated in response to maternal hypoxia (Chen Q. et al., 2014). Furthermore, neuron-specific over-expression of miR-27b in the mouse cortex increased resistance to hypoxia induced apoptosis by inhibiting apoptotic protease-activating factor 1 (Apaf-1) (Chen Q. et al., 2014). Similar observations have been reported in rat primary embryonic hippocampal neuron cultures (Cai et al., 2016) and further potential targets and mechanisms of the miR-27 family have been alluded. For example, miR-27a, directly modulates components of the TLR4 signaling cascade, including TIR domain-containing adaptor molecule-2 (TICAM2) and interleukin-1 receptor-associated kinase 4 (IRAK4), cytoplasmic proteins that link TLR4 and recruit to adaptor protein MyD88 following TLR4 activation, respectively, and coordinates gene transcription and inflammation (Li et al., 2015; Lv et al., 2017). Prophylaxis treatment with miR-27a mimics in an ischemic reperfusion model results in reduced mRNA and protein expression of TICAM2 accompanied by attenuation of TLR4 activation and pro-inflammatory cytokine production, while pretreatment with miR-27a inhibitory oligonucleotides show opposite effects (Li et al., 2015). Comparable anti-inflammatory effects of miR-27a have also been observed in cultured neonatal microglial cells which were achieved by targeting IRAK4 and TLR4 (Lv et al., 2017). Speculatively, miR-27a can target multiple genes and regulate the TLR4 signaling pathway to prevent an excessive inflammatory response to injury. Indeed, various animal models of perinatal brain injury have shown that TLR4 activation and the ensuing inflammatory response can result in cell death and a pattern of injury similar to that seen in human infants, including hypomyelination, glia activation and disruption of thalamocortical function (Dean et al., 2011; Kannan et al., 2011; Dhillon et al., 2015). Therefore, the anti-apoptotic and anti-inflammatory effects of miR-27a/b may prove to be a potential therapeutic target in the future.

Cerebral angiogenesis is a critical reparative process of the microvasculature following hypoxic-ischemic injury, involving cellular cross-talk through neurotropic factors, improving regional blood supply, and facilitating the migration of neurons toward damaged regions (Ohab et al., 2006; Yin et al., 2015). Modulating this reparative process holds promise since the perinatal brain has the greatest potential for repair and recovery (Dzietko et al., 2013). miR-15a in vascular endothelial cells has emerged as a key regulator of angiogenesis, such that downregulation of miR-15a promotes vasculogenesis by increasing neurotrophic factors, including fibroblast growth factor 2 (FGF2) and vascular endothelial growth factor (VEGF) (Yin et al., 2012). Critically, VEGF released by angiogenic endothelial cells can promote proliferation and differentiation of neural progenitor cells via vascular endothelial growth factor receptor 2 (VEGFR2) (Teng et al., 2008). FGF2 is also an important growth factor involved in neurogenesis and gliogenesis during embryonic and postnatal development (Vaccarino et al., 1999). Crosstalk between cerebral endothelium and oligodendrocytes can promote proliferation and migration of late-stage OPCs through FGF2 (Shindo et al., 2016). Given that delayed treatment with VEGF and FGF2 have proven to be neuroprotective in perinatal models of brain injury (Monfils et al., 2006; Dzietko et al., 2013), miR-15a may be an attractive therapeutic target in the tertiary phase of injury (Fleiss and Gressens, 2012). In fact, it may pose advantages given its ability to target multiple genes in addition to delivering a synergistic effect.

MSCs have been extensively applied in both experimental and clinical settings of CNS diseases owing to their immunomodulatory, regenerative and reparative properties including stroke (Koh et al., 2008; Steinberg et al., 2016), multiple sclerosis (Zhang et al., 2005; Gerdoni et al., 2007; Uccelli et al., 2011) and perinatal brain injury (van Velthoven et al., 2010; Jellema et al., 2013; Drommelschmidt et al., 2017). Currently, it is proposed that MSCs exert their therapeutic potency at least in part through a paracrine mechanism involving the release of extracellular vesicles, which based on their size and intracellular origin include microvesicles (∼100–1000 nm in diameter) and exosomes (∼40–100 nm in diameter) (Hass and Otte, 2012; Mokarizadeh et al., 2012; Xin et al., 2012; Lee et al., 2013; Koniusz et al., 2016). Indeed, MSCs are prolific producers of extracellular vesicles; a feature that is maintained with immortalization of cells to generate permanent cell lines, making them an ideal option for biological tissue replacement regeneration (Yeo et al., 2013).

MSC-derived extracellular vesicles are enriched with a variety of proteins and different RNA species (mainly mRNA and miRNA) as well as trophic factors whose functions are linked to MSCs biological effects. Importantly, evidence now suggests that specific miRNAs are necessary to mediate MSC-derived extracellular vesicles neuroprotective effect (Xin et al., 2012). While miRNAs encapsulated within MSC derived microvesicles are predominantly in their precursor form (pre-miRNAs) (Chen et al., 2009), studies have demonstrated the presence and biological functional roles of exosomal mature miRNAs (Koh et al., 2010; Katakowski et al., 2013; Ono et al., 2014). The transfer of miR-133b from exosomal MSCs directly enhanced neurite outgrowth and functional recovery in adult stroke models (Xin et al., 2012, 2013). Given the putative occurrence of impaired neurite outgrowth in perinatal brain injury (Robinson et al., 2006; Dean et al., 2013), exosomal miR-133b may be important for brain connectivity and function. In an elegant series of studies conducted by Xin et al. (2012), miR-133b was substantially downregulated in the ischemic rat brain and increased following MSC intravenous administration. Connective tissue growth factor (CTGF) and RhoA are both inhibitors of neurite growth and are selective targets of miR-133b (Xin et al., 2014). Critically, administration of MSC-derived exosomes enriched with miR-133b reduced CTGF and RhoA expression and exhibited enhanced axonal plasticity, neurite remodeling and functional recovery compared to naturally occurring MSC-derived exosomes (Xin et al., 2013). These changes were confirmed in primary cultured neurons and astrocytes (Xin et al., 2013). Transfer of miR-133b enriched MSC derived exosomes in cultured neurons, inhibited RhoA expression and stimulated neurite outgrowth, while the transfer in astrocytes, downregulated CTGF expression, a known inhibitor of axonal growth and contributor to glial scar formation in human cerebral infarction (Schwab et al., 2000; Xin et al., 2013).

In a recent conducted study by Yang et al. (2017), rabies virus glycoprotein modified exosomes were employed to achieve neuron-specific delivery of miR-124 across the blood–brain barrier. Previously, invasive cerebral administration of miR-124, a regulator of neurogenesis (Makeyev et al., 2007; Cheng et al., 2009; Åkerblom et al., 2012), was reported to reduce infarct area and improve neuronal survival against ischemic injury in mice (Liu et al., 2011; Doeppner et al., 2013). In the study conducted by Yang et al. (2017), rabies virus glycoprotein exosomes effectively carried miR-124 to neurons of the ischemic region and supported neuronal identity of cortical neural progenitors and reduced ischemic cortical injury by robust neurogenesis. Thus the above evidence supports the therapeutic potential to ameliorate neuronal injury by exploiting the neurodevelopmental function of miRNAs.

Additionally, in concordance with Xin et al. (2012, 2013), MSC derived exosomes provide a therapeutically viable delivery of gene drugs to the brain and possibly specific cells across the blood–brain barrier. Since MSCs produce an abundant source of extracellular vesicles that contain a selection of miRNAs with the potential to elicit neuroprotective biological processes in response to injury, including the ability to modulate the action of neighboring cells, it seems worthwhile to investigate whether MSC-extracellular vesicles would be a promising therapy to promote neurological functional recovery in the developing brain following injury. Indeed, recent *in vivo* investigations using animal models of perinatal and neonatal brain injury support their application (Drommelschmidt et al., 2017); however, further investigations are required to characterize what specific miRNA profiles potentially contribute to protection.

Finally, it is pertinent to mention that exosomes/extracellular vesicles, viewed as potent vehicles by which to deliver potentially therapeutic miRNAs to the brain, can equally participate in the pathophysiological processes of blood-CSF-brain-communication. Recent studies undertaken in both an *in vivo*, *in vitro*, and *ex vivo* mouse model of endotoxemia have shown that miRNA-containing extracellular vesicles originating from the choroid plexus epithelium can enter brain parenchymal cells and via astrocytic and microglial processes induce miRNA target repression and inflammatory gene expression (Balusu et al., 2016). The transfer of potentially adverse proinflammatory driven extracellular vesicle-derived miRNAs to the brain via this route of communication would seem of considerable importance for the advancement of our understanding of the pathophysiological mechanisms of intrauterine infection-related preterm brain injury (Dammann and Leviton, 1997; Malaeb and Dammann, 2009), including the role of placental vesicle-derived miRNAs (Ilekis et al., 2016; Wei et al., 2017; Salomon et al., 2018) in this process and warrants further investigation.

## Conclusion and Future Directions

In the past decade, numerous articles have been published on the role of miRNAs within the brain. As post-transcriptional regulators of gene expression, miRNAs most definitely play a crucial role in the development of the brain. Nevertheless, research conducted to define their impact on the developing and injured brain is still in its infancy. Given specific miRNAs can exhibit diverse functional roles throughout development and can act synergistically, identification of their precise functional roles is fraught with difficulties. This is particularly relevant when considering adopting specific miRNAs as biomarkers of perinatal brain injury. Thus, careful interpretation of data is required not only in the context of biomarker potential, but also application as a therapeutic strategy since off-target effects can confound the latter. One attractive possibility to ensuring, at least targeted delivery, is the fast developing field of research involving exosome-based miRNA therapies for neurological injuries and disorders. Exosomes are considered a key carrier of circulating miRNAs. Since they mediate the exchange of miRNAs between cells, readily cross the blood–brain barrier and fuse with cell membranes, they hold promise not only as a miRNA biomarker carrier, but also as a means to deliver miRNA-based therapies to the developing injured brain. Clearly, continued developments in this field of research has the potential to enhance future prospects of effectively treating perinatal brain injury especially those vulnerable to premature injury.

## Author Contributions

KHTC and MF devised main conceptual ideas and outlines and took the lead in writing the manuscript. All authors contributed to the manuscript and provided feedback and discussed the manuscript.

## Conflict of Interest Statement

The authors declare that the research was conducted in the absence of any commercial or financial relationships that could be construed as a potential conflict of interest.
